# Altered functional connectivity after pilocarpine-induced seizures revealed by intrinsic optical signals imaging in awake mice

**DOI:** 10.1117/1.NPh.11.1.015001

**Published:** 2023-12-19

**Authors:** Lifen Gong, Xin Huang, Zhe Hu, Chen Chen, Ziqi Zhang, Hongxuan Liao, Yinglin Xiao, Jianchen Fan, Linghui Zeng, Shangbin Chen, Yicheng Xie

**Affiliations:** a Children’s Hospital, Zhejiang University School of Medicine, National Clinical Research Center for Child Health, Department of Neonatal Surgery, Hangzhou, China; b The First Affiliated Hospital, Zhejiang University School of Medicine, Department of Neurosurgery and Pediatrics, Hangzhou, China; c Huazhong University of Science and Technology, Britton Chance Center for Biomedical Photonics, Wuhan National Laboratory for Optoelectronics, Wuhan, China; d Hangzhou City University, School of Medicine, Key Laboratory of Novel Targets and Drug Study for Neural Repair of Zhejiang Province, Hangzhou, China

**Keywords:** seizures, intrinsic optical signal, cortical functional network, default mode network, awake

## Abstract

**Significance:**

Comorbidities such as mood and cognitive disorders are often found in individuals with epilepsy after seizures. Cortex processes sensory, motor, and cognitive information. Brain circuit changes can be studied by observing functional network changes in epileptic mice’s cortex.

**Aim:**

The cortex is easily accessible for non-invasive brain imaging and electroencephalogram recording (EEG). However, the impact of seizures on cortical activity and functional connectivity has been rarely studied *in vivo*.

**Approach:**

Intrinsic optical signal and EEG were used to monitor cortical activity in awake mice within 4 h after pilocarpine induction. It was divided into three periods according to the behavior and EEG of the mice: baseline, onset of seizures (onset, including seizures and resting in between seizure events), and after seizures (post, without seizures). Changes in cortical activity were compared between the baseline and after seizures.

**Results:**

Hemoglobin levels increased significantly, particularly in the parietal association cortex (PT), retrosplenial cortex (RS), primary visual cortex (V1), and secondary visual cortex (V2). The network-wide functional connectivity changed post seizures, e.g., hypoconnectivity between PT and visual-associated cortex (e.g., V1 and V2). In contrast, connectivity between the motor-associated cortex and most other regions increased. In addition, the default mode network (DMN) also changed after seizures, with decreased connectivity between primary somatosensory region (SSp) and visual region (VIS), but increased connectivity involving anterior cingulate cortex (AC) and RS.

**Conclusions:**

Our results provide references for understanding the mechanisms behind changes in brain circuits, which may explain the profound effects of seizures on comorbid health conditions.

## 1 Introduction

Seizures are uncontrolled electrical disturbances in the brain, often linked to epilepsy.^1^ The cause of seizures varies but can be summarized by anything that disrupts neuron communication in the brain.^2^ A seizure often lasts for a short duration, from 30 s to 2 min, most of which can be controlled with medication. However, a seizure that lasts longer than 5 min is still a medical emergency.^3^ No matter the duration, seizures can end with long-lasting behavioral changes in movements, feeling, consciousness, and cognition. Moreover, comorbidities, particularly psychological problems, exist after seizure onset, indicating a prolonged brain circuit impairment induced by seizures that can place a high burden on patients, families, and society.^4^

Epilepsy is a network disorder characterized by recurring seizures that extend beyond the epileptic focus to impact other areas of the brain.^5^ In clinics, functional magnetic resonance imaging (fMRI) and electroencephalography (EEG) recording have been performed in epileptic patients, where they found altered activity and network connectivity in the brain,^6^ particularly the alterations in the default mode network (DMN), and the resting state functional connectivity network of the dorsal attention and executive networks.^7^^,^^8^ However, enhanced or diminished network connectivity remains controversial due to subject variability.^9^^–^^11^ Recently, the propagation and network changes have been studied using fMRI in a kindling model induced by stimulation of the ventral hippocampus in rats, where increased brain-wide activity and bilateral functional connectivity were found during seizures.^12^ But it was performed after recurrent seizures were formed and sedation during the imaging may confound the results and interpretation.^13^ In addition, due to the limited temporal and spatial resolution of brain activity and network recordings (fMRI, EEG), their network changes are not clear in clinical and animal model studies.^14^^,^^15^ To explain the signs and symptoms induced by seizures, it is crucial to improve detection resolution and to detect changes in functional connectivity post seizures in awake status.

Here, our study investigated the impact of seizures on brain activity and network connectivity in awake mice by using *in vivo* mesoscale imaging of intrinsic optical signal (IOS)^16^ and EEG. IOS measures brain activity through neurovascular responses, specifically by analyzing changes in the intensity of light reflected from the cortical surface and translating them into changes in local hemoglobin content.^17^ This approach parallels the methodology used in fMRI.^18^ Noticeably, as a simple bench-top method to perform brain imaging and functional mapping of the cortex in mice, IOS consists of the features of genetic manipulation free, high resolution and speed, and at a low cost.^19^ By capitalizing on rest-state activity, large-scale functional networks with the topographical organization were revealed using IOS. In addition, IOS was performed with relatively non-invasive transparent craniotomy and head-fixing apparatus in awake mice and is easily amendable to integrate with EEG.^20^^,^^21^ For this study, a pilocarpine (PILO) induced seizure model was chosen due to its widespread use in generating seizures in mice and its convenience to be applied through intraperitoneal injection.^22^

In this study, we found hemoglobin content was changed after seizure induction and that it progressively, but only partly, returned to normal when seizures ended. The hemoglobin content in the parietal association cortex (PT), retrosplenial cortex (RS), primary visual cortex (V1), and secondary visual cortex (V2) was affected the greatest. We also observed changes in functional connectivity, with decreased connectivity between the visual association regions and PT. In contrast, functional connectivity increased between the motor association regions and most other regions. In addition, seizures led to considerable alterations in the DMN, a network critical for sustaining normal brain activity and higher cognitive function. Specifically, we observed hypoconnectivity between primary somatosensory region (SSp) and visual region (VIS), while hyperconnectivity between anterior cingulate cortex (AC)/RS and other regions. Our findings suggest that seizures induce patterned changes in cortical activity and functional networks. We propose IOS may be a valuable tool for detecting changes in cortical activity and functional networks and providing a circuit foundation for brain dysfunctions in network disorders in an animal model of seizures.^23^^,^^24^

## 2 Materials and Methods

### 2.1 Animals

**Fig. 1:**
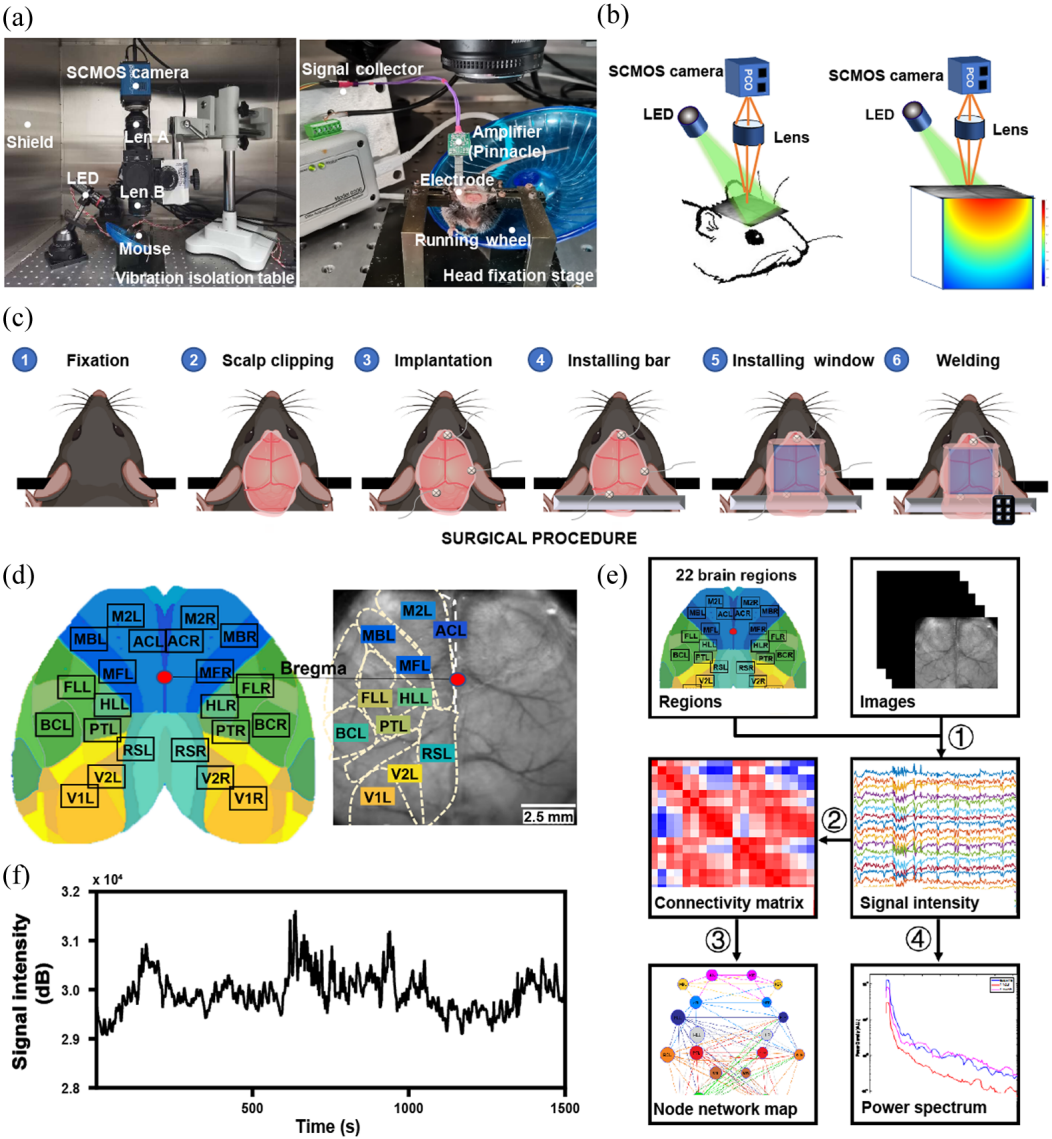
The system and analytical procedures for IOS imaging and EEG recording in head-fixing awake mice. (a) Instruments of IOS system (left) and EEG signal acquisition (right). (b) The schematic diagram of IOS imaging. (c) Surgical procedures of window installation and electrode implantation. (d) Based on the Allen Brain Mouse Atlas, a schematic of the bilateral craniotomy shows the ROI in the cortex. (e) Imaging analysis flow chart. ①Signal changes over time were extracted from the preprocessed images. ②A connectivity matrix was obtained by calculating the Pearson correlation coefficient between each pair of regions. ③A new matrix removed the weakest connectivity by applying to the threshold is visualized as a network. Lower spectrum of raw signal changes over time was analyzed. (f) A time series of the raw signal changes over time in MBL. IOS, intrinsic optical signal; ROI, regions of interest; MB, whisker-associated motor cortex; L, left; R, right.

All animal experiments were performed in accordance with institutional guidelines and were approved by Zhejiang University Animal Care. C57BL/6 mice were anesthetized and placed on a heating pad maintained at 37°C. The head of a mouse was secured in a stereotactic frame using a nose clip and ear bars. The scalp fur was shaved, a midline incision was made along the top of the head, and the scalp was carefully removed to expose the skull, which measures $∼1\;{\mathrm{cm}}^{2}$. For cortical EEG recordings, an electrode was implanted on the right side of the mouse (AP: −4.0  mm; ML: 1.9 mm; DV: −0.5  mm). Two screws were placed on the superior and inferior sides of the cerebellum’s skull to serve as a reference and a ground electrode, respectively. The leads were made of copper wire and attached to the rows of pins. A $1\;{\mathrm{cm}}^{2}$ sized window was made from a 0.13 mm thick piece of glass and fixed to the skull with clear dental adhesive. Mice were rested for 1 week after surgery to ensure that they could move freely. To adapt the mice to the experimental environment, we placed the mouse on a running wheel device for acclimatization for 30 min each time, three times a day for 1 week [Fig. 1(c)]. Subsequently, nine 8-week-old male C57BL/6 mice (22.33±1.52  g) that had survived surgery and were able to move freely, were bred in the Laboratory Animal Center of Zhejiang University. They were housed at a constant temperature of 22±2°C with a 12-h light/dark cycle and free access to food and water.

### 2.2 Anesthesia Model

Mice were injected intraperitoneally with pentobarbital at a dose of 40  mg/kg. Images were captured 30 min post-injection, during which the mice exhibited immobile limbs, and EEG recordings revealed suppressed brain activity.

### 2.3 Seizure Model

PILO model: mice were injected intraperitoneally with PILO (P6503-5G, Sigma) at a dose of 350  mg/kg. The seizure grade was assessed according to the Racine score and EEG. Racine score: grade I: facial muscle spasm and twitching; grade II: spasms of neck muscles; grade III: clonus of one forelimb; grade IV: bilateral forelimb spasms; grade V: inability to stand, loss of balance, twitching of limbs. If the mice did not develop grade IV seizure or lasting spike-wave on EEG within 30 min, they were excluded from the subsequent study.

### 2.4 IOS Imaging

To ensure the stability of the imaging system, an anti-vibration table was used. The metal shielding box was inverted on the table to help shield the imaging system from external light and the environment. We have chosen a high-frequency, high signal-to-noise ratio sCMOS camera (pco.edge, PCO, DEU) with lenses (50 mm, f1:1.8 d: 85 mm, f1.8). The focal length ratio of the upper and lower lenses determines the magnification of the mouse window by 1.6 times in this experiment. Microscopic imaging was formed by serial connection, and the field of imaging was $∼10\times 10\;{\mathrm{mm}}^{2}$, including the entire cortex. The microscope base was used to hold the imaging lens in a fixed overhang [Fig. 1(a)]. The green LED light (530 nm) source was directed to the imaging glass surface, and the wavelength of light was absorbed and reflected by the red blood cells in the brain vessels, and the reflected light was received by the sCMOS camera [Fig. 1(b)]. The sampling frequency was 10 Hz and the acquisition lasted for 5 min followed by a 16-bit image of 512×512  pixels. Sixteen-bit images have better photon capacity, higher signal-to-noise ratio, and can use lower frequency to acquire images and reduce the data processing effort. Then, binning at 4:4:2 (x:y:z) was performed on the ImageJ to reduce the output image size from 512×512  pixels to 128×128  pixels, from 3000 frames to 1500 frames [Fig. 1(e)]. Imaging frequency: after PILO injection images were taken every 10 min for the first 30 min, then every 30 min for the next 4 h. It was divided into three periods according to the behavior and EEG of the mice: baseline, onset (including seizures and resting within seizures), and post (after seizures).

### 2.5 Imaging Analysis

The selection of all ROIs was guided by comparison with the Atlas. Seed images were obtained by comparing the acquired images with the Histological Atlas (Paxinos and Franklin, 2012), and a total of 8 ROIs were defined in the left and right hemispheres, including somatosensory, motor, posterior splanchnic, and visual cortex regions. For resting state images, we further extended to 22 regions of interest (ROIs) based on the Allen Brain Atlas.^25^ The mean signal intensity was first calculated for each region. A point in the center of the region of interest was selected as the seed, and four points are selected above and below the seed, for a total of five seeds selected to represent the region. The average signal intensity change was calculated to reflect the change in hemoglobin level. For accurate comparisons, images of each mouse were aligned, as determined by two anatomical landmarks, the bregma, and the retrogma. All subsequent comparisons were performed after all mice were aligned.

### 2.6 Functional Connectivity

Functional connectivity: The temporal signal at each pixel point was extracted using MATLAB (R2021a, MathWorks). As mentioned above, five representative seeds were identified in each region, and the time-signal correlation between the seeds in other regions and the representative seeds was calculated. Finally, the correlation of each region at different times was plotted. In addition, the 22 ROIs were extended. Consistently, the temporal trajectories of five seeds were correlated with seeds in other regions to create a 22×22 connectivity matrix [Figs. 1(d) and 1(e)]. In short, connectivity between two regions was measured by the Pearson correlation coefficient.^16^ The equation is as follows:

$$\rho X,Y=\frac{\sum_{n=0}^{N-1}(X[n]-\mu_{X})(Y[n]-\mu_{Y})}{(N-1)\sigma X\sigma Y}.$$

**Fig. 2:**
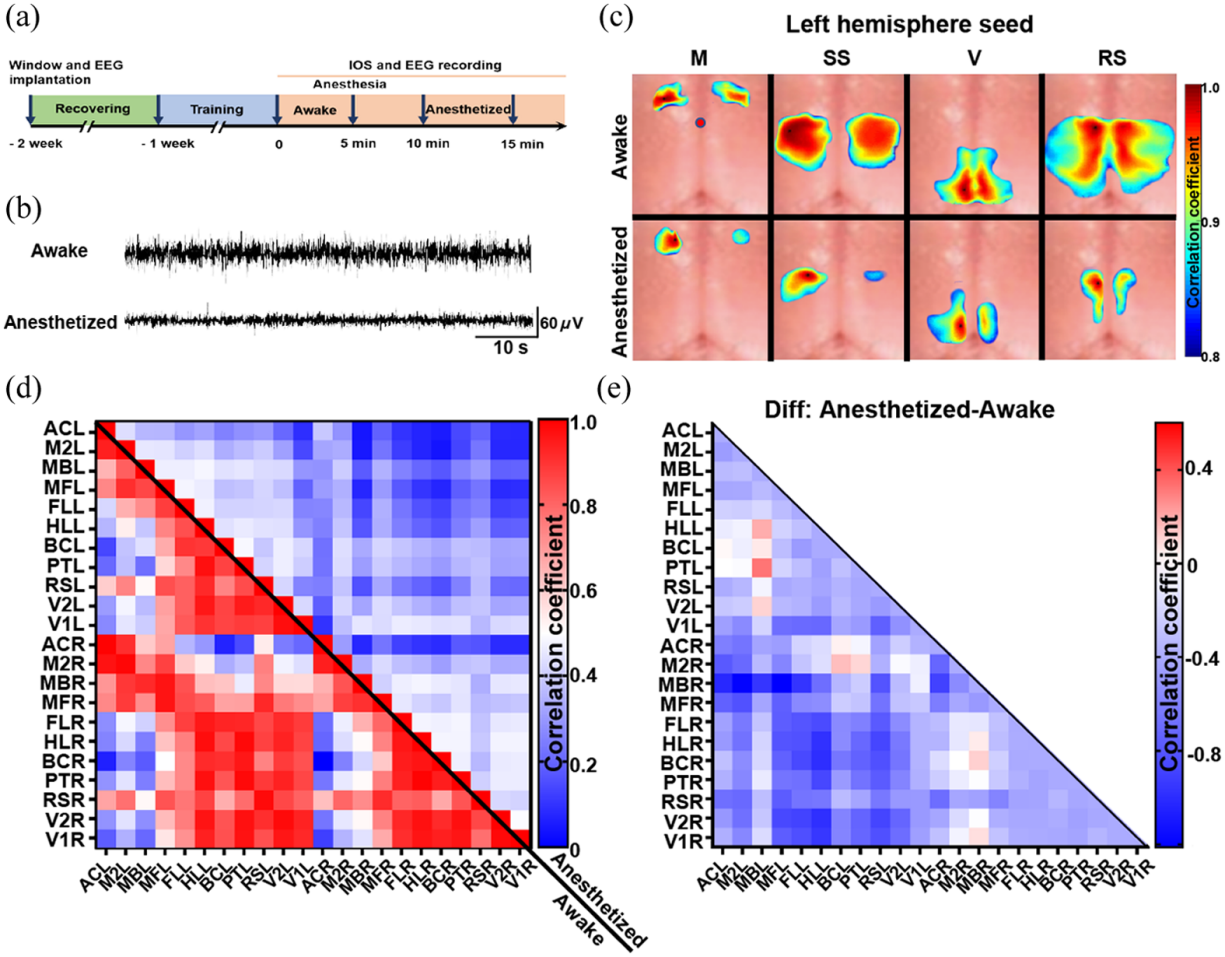
Anesthesia induces a widespread suppression of functional connectivity. (a) The design of the experiment. (b) Representative EEG at awake and anesthetized states was recorded in the mouse cortex. (c) Representative functional connectivity brain maps are shown for awake and anesthetized mice, with seeds placed in the left hemisphere. Interhemispheric functional connectivity is significantly decreased in the M, SS, V, and RS in anesthetized mice. (d) The functional connectivity matrix from IOS imaging shows significant differences between the awake (lower half) and the anesthetized state (upper half). (e) The difference matrix was calculated by subtracting the awake matrix from the anesthetized matrix, which shows a widespread suppression of functional connectivity in anesthetized mice. IOS, intrinsic optical signal; M, motor cortex; SS, somatosensory cortex; V, visual cortex; RS, retrosplenial cortex; L, left; R, right.

The signal intensity data for the two regions are X and Y, respectively. The μ is defined as mean signal intensity and σ is the standard deviation.

### 2.7 EEG Parameter Settings

The EEG acquisition system parameters were set to bandpass filtering 1 to 1000 Hz, a sampling frequency of 1000 Hz, and a signal amplifier of 100×.

### 2.8 Graph Theoretical Measures

The Bioinformatics Toolbox of MATLAB was used to output a network diagram of the functional connectivity. The nodes in the graph represent different brain regions, and the temporal correlation of activity in different brain regions represents functional connectivity. Further, we discarded the spurious connections with less strength by setting the threshold value. The optimal network graph was drawn by comparative analysis of network graphs made under different thresholds [Fig. 1(e)].

### 2.9 Power Density Measures

The images at baseline and 2 h after seizures were converted into three-dimensional matrices and stored in two datasets, respectively. Power density in the two states in each brain region of each mouse was made by using reshape, the pmtm function, averaging, etc. The data sampling number of the above waveform diagram was first determined to be 1000, the 0.00 to 0.05 Hz and 0.05 to 0.10 Hz were converted to sampling numbers, then the resulting three-dimensional matrices were used to calculate the average spectral energy at baseline, 2 h after seizures per brain region for each mouse using the mean function [Fig. 1(e)].

### 2.10 Statistical Analysis

Statistical analysis was performed using GraphPad Prism 8.0 (GraphPad Software Inc., San Diego, California). Data were expressed as the mean ± SD. *p<0.05 was considered to be significant. All data were tested for normal distribution using the Shapiro–Wilk test. Comparisons between multiple experimental groups were made using paired one-way ANOVA with Tukey’s post hoc test. For comparisons between paired two groups, paired t-test was used. If the data were not normally distributed, the Wilcoxon test was used.

## 3 Results

### 3.1 Functional Network in the Mouse Cortex Revealed by IOS Imaging Is Extensively Suppressed During Anesthesia

Animals are often anesthetized in the majority of studies for the functional network at rest. But the impact of anesthesia on these networks is often neglected. To address this gap, we combined EEG and IOS to examine alterations in the functional network in the mouse cortex during both awake and anesthetized states [Fig. 2(a)]. Our EEG recording revealed that the amplitude of the spikes was lower during anesthesia than during the awake state, indicating suppression of cortical neuronal activity [Fig. 2(b)]. During anesthesia, both the ipsilateral and contralateral hemispheres, including the motor cortex (M), the somatosensory cortex (SS), visual cortex (V), and RS, exhibited widespread decreases in functional connectivity [Fig. 2(c)]. To further investigate these findings, we subdivided the functional regions according to the Allen Brain Mouse Atlas, plotted the matrices [Fig. 2(e)] and created the difference matrix [Fig. 2(f)], which also confirmed overall decreases in functional connectivity in the cortex during anesthesia. In conclusion, anesthesia significantly suppresses functional connectivity of the functional network in the mouse cortex.

### 3.2 Raw Signals of IOS Imaging in the Cortex Are Suppressed After Seizures

**Fig. 3:**
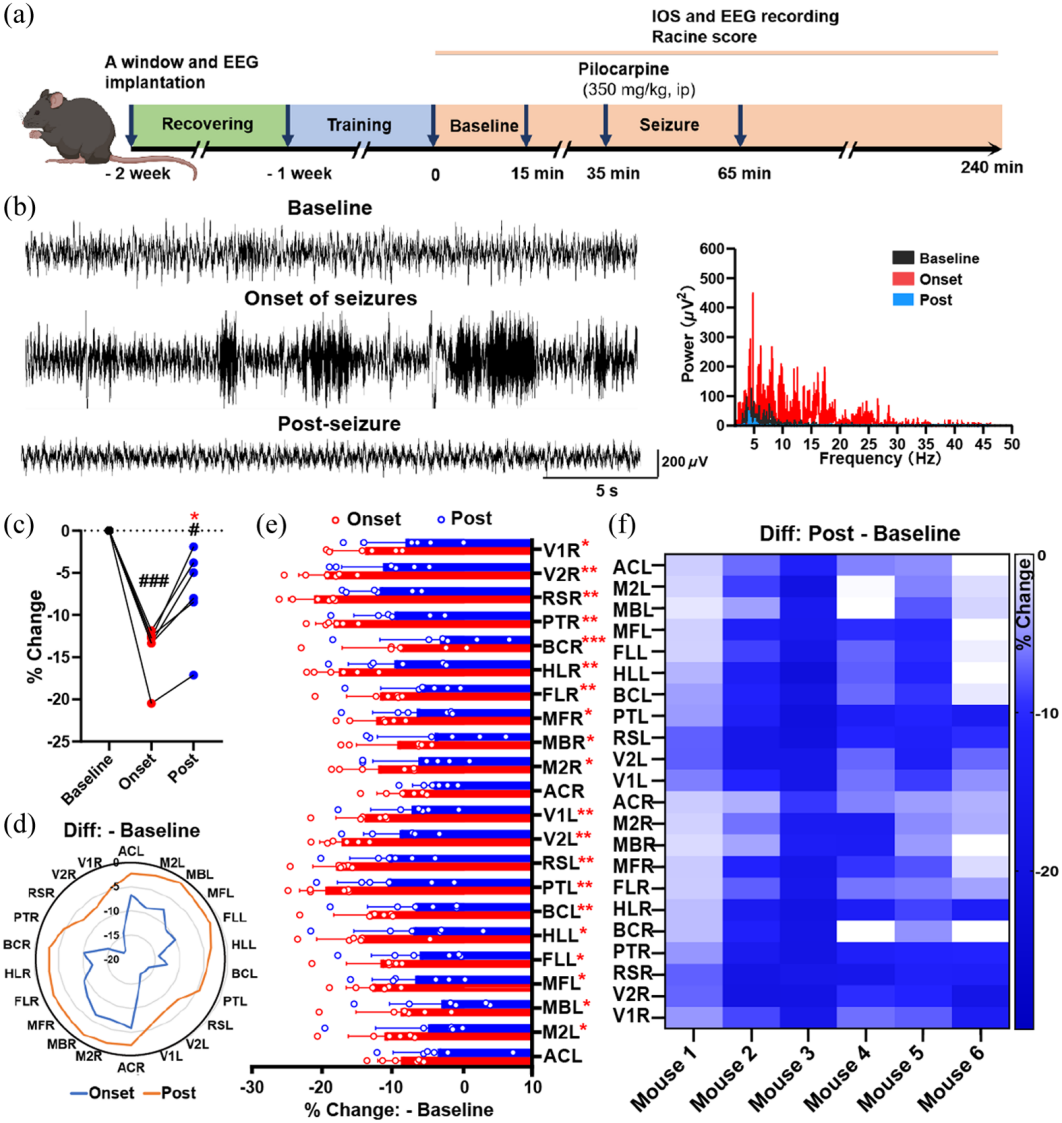
The raw signals of IOS imaging in the cortex are suppressed after seizures. (a) The design of the experiment. (b) Representative EEGs (left) during the baseline (upper), the onset of seizures (middle), and post-seizures (bottom) were recorded in the cortex in a PILO-induced mouse model. The corresponding spectrum (right) was analyzed by Pinnacle Technology’s Sirenia Seizure Pro software. Based on the EEG spectrum, seizures are characterized by the appearance of high-amplitude, high-frequency activity. The spikes in the post group are characterized by lower power than the baseline period. (c) The scatter plot of the mean signal changes shows significant decreases during the onset and post-periods without seizure activity. n=6. (d) The representative map depicts the 22 regions of mean signal intensity changes in a mouse, which shows a significant decrease in the onset or post group compared to the baseline. (e) The statistical plot of the 22 regions of mean signal intensity changes shows a significant decrease in the onset or post period compared to the baseline, particularly in the PT, RS, V2, and V1. n=6. (f) A map of changes in the 22 regions of mean signal intensity shows a reduction after seizures in each mouse. n=6. Data are presented as standard deviation. *p<0.05, **p<0.01, ***p<0.001, paired two-tailed Student’s t-test. PT, parietal association cortex; RS, retrosplenial cortex; V2, secondary visual cortex; V1, primary visual cortex; L, left; R, right.

In our study, brain activity was recorded by both IOS and EEG during baseline, onset of seizures (onset, including seizures and resting among seizure events), and after seizures (post, without seizures) [Fig. 3(b)]. The mean raw signals extracted from IOS imaging showed a significant decrease during the onset of seizures compared to the baseline, indicating an increase in hemoglobin content and enhanced cortical neuronal activity [Fig. 3(c); baseline versus onset, 0.00±0.00% versus −14.03±1.31%, ***p<0.001]. This was consistent with the neuronal activity recorded by EEG [Fig. 3(b)]. After seizures, the mean signals increased significantly, indicating a recovery of hemoglobin content, but still did not return to the baseline by the end of the observation time [Fig. 3(c); onset versus post: −14.03±1.31% versus −7.38±2.20%, **p<0.01; baseline versus post, 0.00±0.00% versus −7.38±2.20%, ^#^p<0.05]. Moreover, EEG revealed postictal generalized suppression post seizures [Fig. 3(b)]. These findings suggest that even though neuronal electrical activity is decreased after seizures, the brain maintains a high metabolic rate, which is reflected in the increased hemoglobin content. Furthermore, upon subdividing the functional areas, a noteworthy rise in hemoglobin content during and after seizures compared to baseline was observed in the PT, RS, V2, and V1 regions [Figs. 3(d) and 3(e)]. This may indicate that these regions are susceptible to change post seizures.

### 3.3 Low-Frequency Oscillatory Signals in the Cortex Are Suppressed After Seizures

**Fig. 4:**
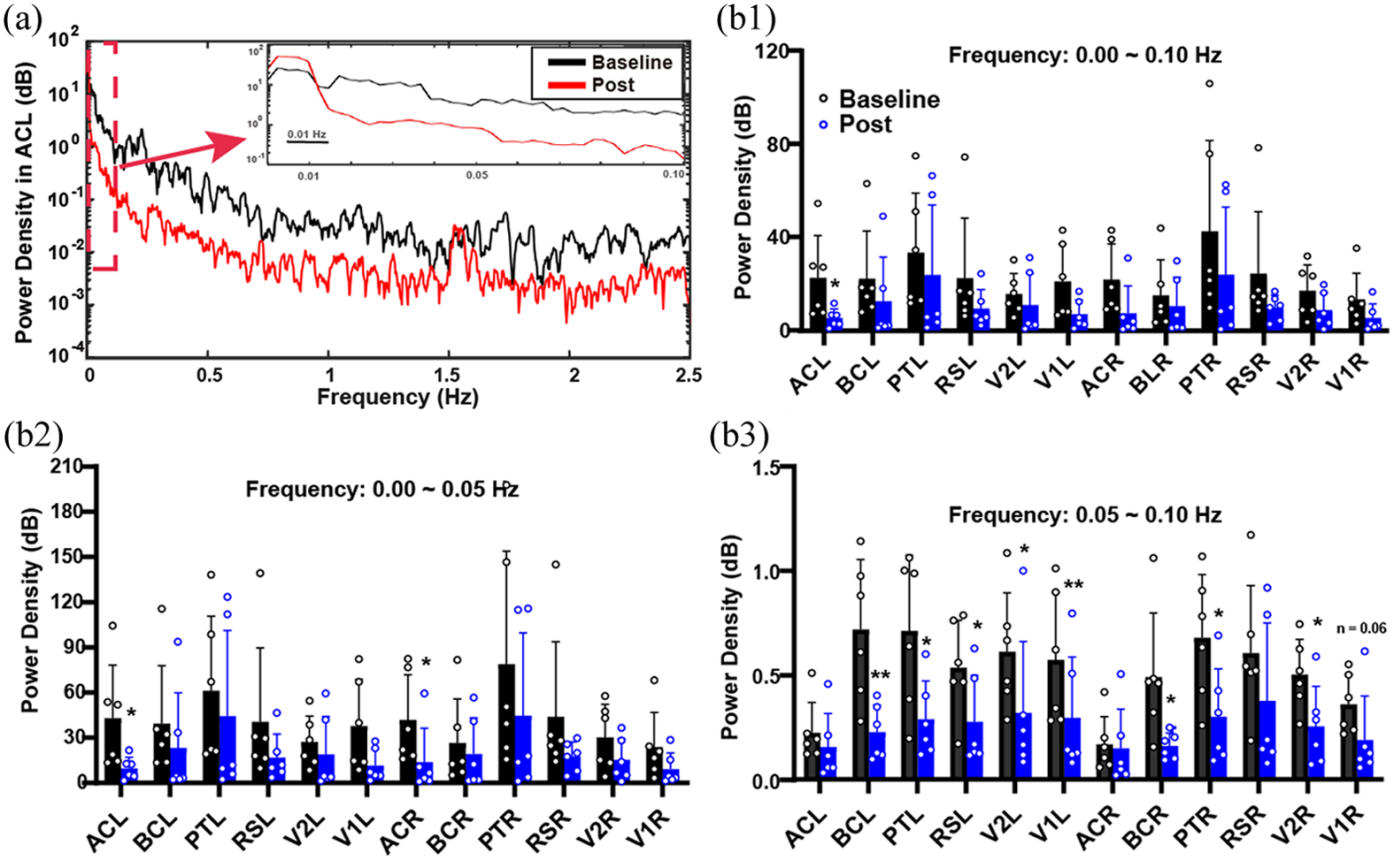
Low-frequency oscillatory signals in the cortex are suppressed after seizures. (a) The representative power spectrum of the low-frequency hemodynamic fluctuations and amplified power spectrum in the 0.00 to 0.10 Hz band in the ACL region. (b) The histograms show the average spectrum energy across three bands, 0.00 to 0.10 Hz (b1), 0.00 to 0.05 Hz (b2), and 0.05 to 0.10 Hz (b3) in the baseline and post-seizures. The average power of the right and left AC regions shows significant decreases post-seizures compared to the baseline in the 0.00 to 0.10 and 0.00 to 0.05 Hz bands. The average power of the left RS and V1, the right and left PT, BC, and V2 shows significant decreases post-seizures in the 0.05 to 0.10 Hz band. Data are presented as mean ± standard deviation. *p<0.05, **p<0.01, ***p<0.001, paired two-tailed Student’s t-test. RS, retrosplenial cortex; AC, anterior cingulate cortex; PT, parietal association cortex; V1, primary visual cortex; BC, primary barrel cortex; V2, secondary visual cortex; L, left; R, right.

Power spectrum analysis of oscillatory signals (0.1 Hz) from 22 cortical brain regions was performed to observe the spatial distribution of spontaneous activity [Fig. 4(a)]. The mean power in the AC of both hemispheres decreased significantly after seizures [Fig. 4(b1); baseline versus post, ACL: 22.48±18.21  dB versus 5.22±3.90  dB, *p<0.05; ACR: 21.74±15.35  dB versus 7.27±11.83  dB, *p<0.05]. Further analysis of the 0 to 0.05 Hz bands and 0.05 to 0.1 Hz bands showed that the average power in AC region decreased significantly only in the 0.00 to 0.05 Hz bands after seizures [Fig. 4(b2); baseline versus post, ACL: 42.71±35.34  dB versus 9.65±7.36  dB, *p<0.05; ACR: 41.75±30.12  dB versus 13.68±22.68  dB, *p<0.05]. While the average power in one side of RS and V1, and both sides of PT, BC, and V2 significantly decreased in the 0.05 to 0.10 Hz band after seizures [Fig. 4(b3)].

### 3.4 Functional Connectivity in the Cortex Is Altered After Seizures

**Fig. 5:**
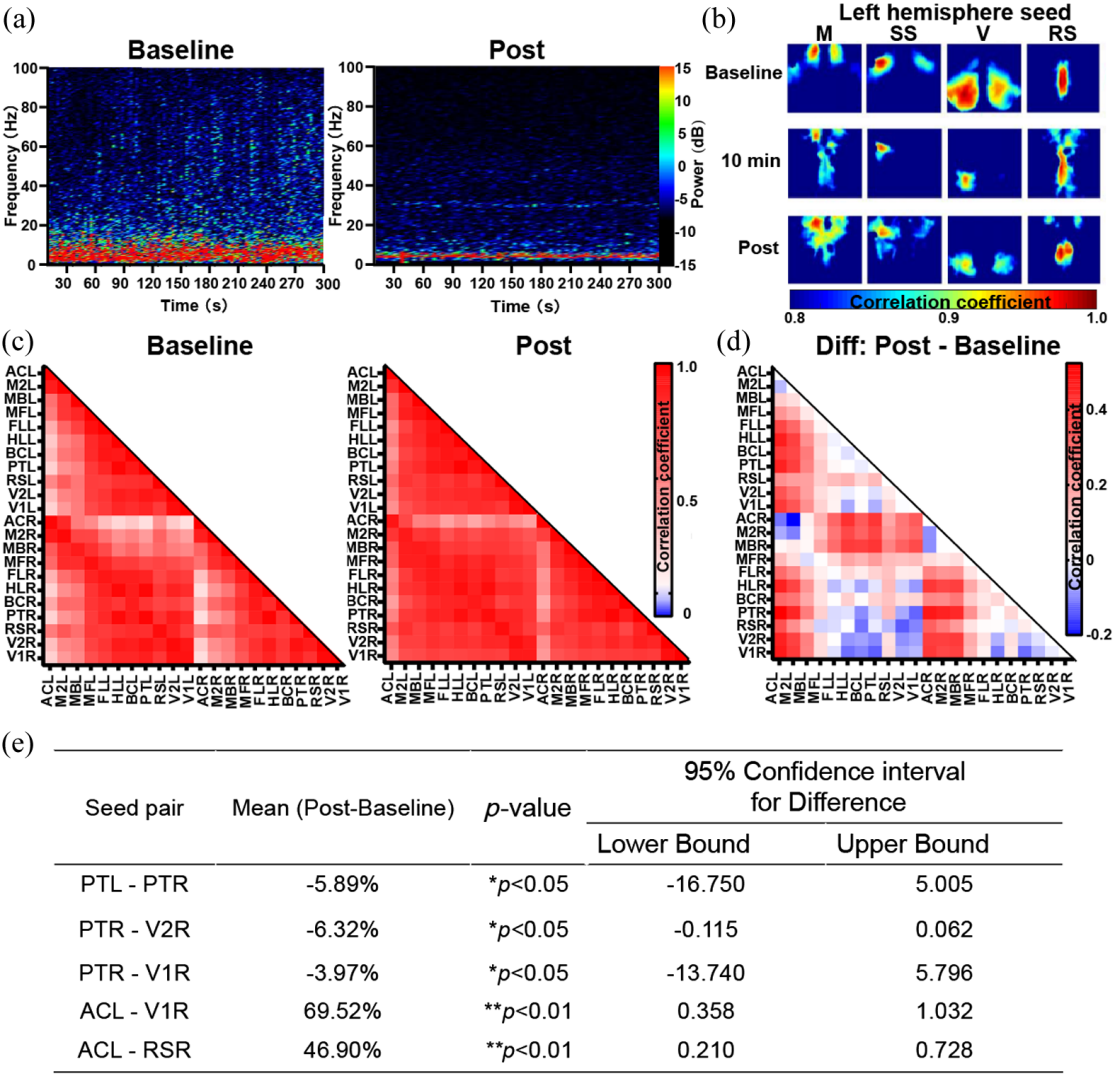
Functional connectivity in the cortex is altered after seizures. (a) Representative heat maps of EEG in the baseline (left) and post (right) periods show suppression of electrical activity after seizures. (b) The representative images show functional connectivity brain maps at different times, with seeds placed in the left hemisphere. The interhemispheric functional connectivity of SS, V significantly decreases after seizures, except in the M. (c) The functional connectivity matrix from IOS imaging shows significant differences between the baseline (left) and the post-seizure (right) period, representing the data from 6 mice. (d) The difference matrix is calculated by subtracting the baseline matrix from the post matrix, representing data from 6 mice. The results indicate decreased connectivity between PT and visual association regions (e.g., V1, V2), as well as between RSR and visual association regions. In contrast, it shows increases in functional connectivity between AC and most other regions, between the M2 and most other regions, as well as between MB and most other regions after seizures. (e) Summary of seed-based interregional functional connectivity. n=6. *p<0.05, **p<0.01, ***p<0.001, paired two-tailed Student’s t-test. IOS, intrinsic optical signal; M, motor cortex; SS, somatosensory cortex; V, visual cortex; RS, retrosplenial cortex; PT, parietal association cortex; V1, primary visual cortex; V2, secondary visual cortex; AC, anterior cingulate cortex; M2, secondary motor cortex; MB, whisker associated motor cortex; L, left; R, right.

**Fig. 6:**
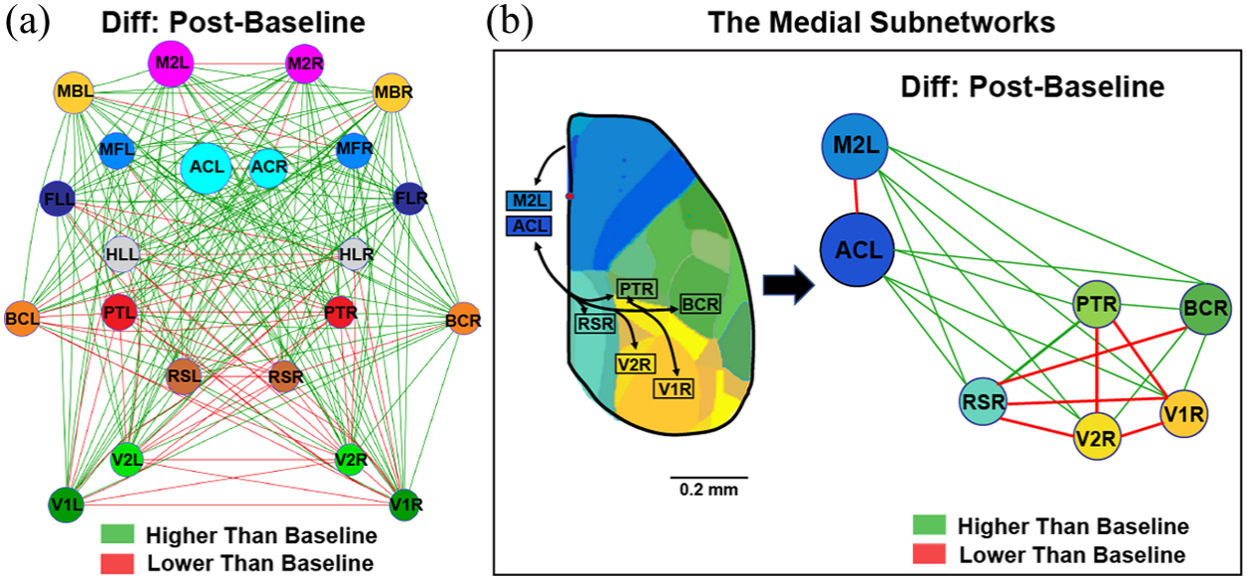
The medial subnetworks are altered after seizures. (a) The mean functional network node map shows significant alterations among the brain nodes (threshold = 0.04). The map shows enhanced connectivity in the front half of the cortex and reduced connectivity in the posterior half cortex. n=6. (b) The map of the medial subnetworks is proposed to indicate information transfer between the sensory regions (e.g., BC, V1, and V2) and higher-order association regions (e.g., AC, RS, and PT). A significantly decreased connectivity between the PTR and PTL, V1R, V2R, while connectivity increased between the ACL and most other regions as well as that between the M2L and most other regions after seizures. n=6. RS, retrosplenial cortex; AC, anterior cingulate cortex; PT, parietal association cortex; V1, primary visual cortex; V2, secondary visual cortex; BC, primary barrel cortex; L, left; R, right.

To investigate the changes in functional networks at rest after seizures, functional connectivity maps were generated from seeds. Results showed reduced connectivity between the ipsilateral and contralateral hemispheres, particularly in the SS and V regions [Fig. 5(b)]. Conversely, functional connectivity was increased between the M in both hemispheres. Then, functional connectivity matrices [Fig. 5(c)] and the difference matrix [Fig. 5(d)] showed decreased connectivity between PT and the visual association regions (e.g., V1 and V2), while connectivity between AC and most other regions, as well as between the motor association regions (e.g., secondary motor cortex, M2, and whisker-associated motor cortex, MB) and most other regions, increased [Fig. 5(d)]. These alterations were significant between PTR and PTL, V1R, V2R, as well as between ACL and V1R, RSR, after seizures [Fig. 5(e)], all of which belong to the medial subnetwork [Figs. 6(a) and 6(b)], suggesting a prolonged impact on information transfer between sensory and higher-order association regions.^26^

### 3.5 PILO Model Alters DMN in the Cortex After Seizures

**Fig. 7:**
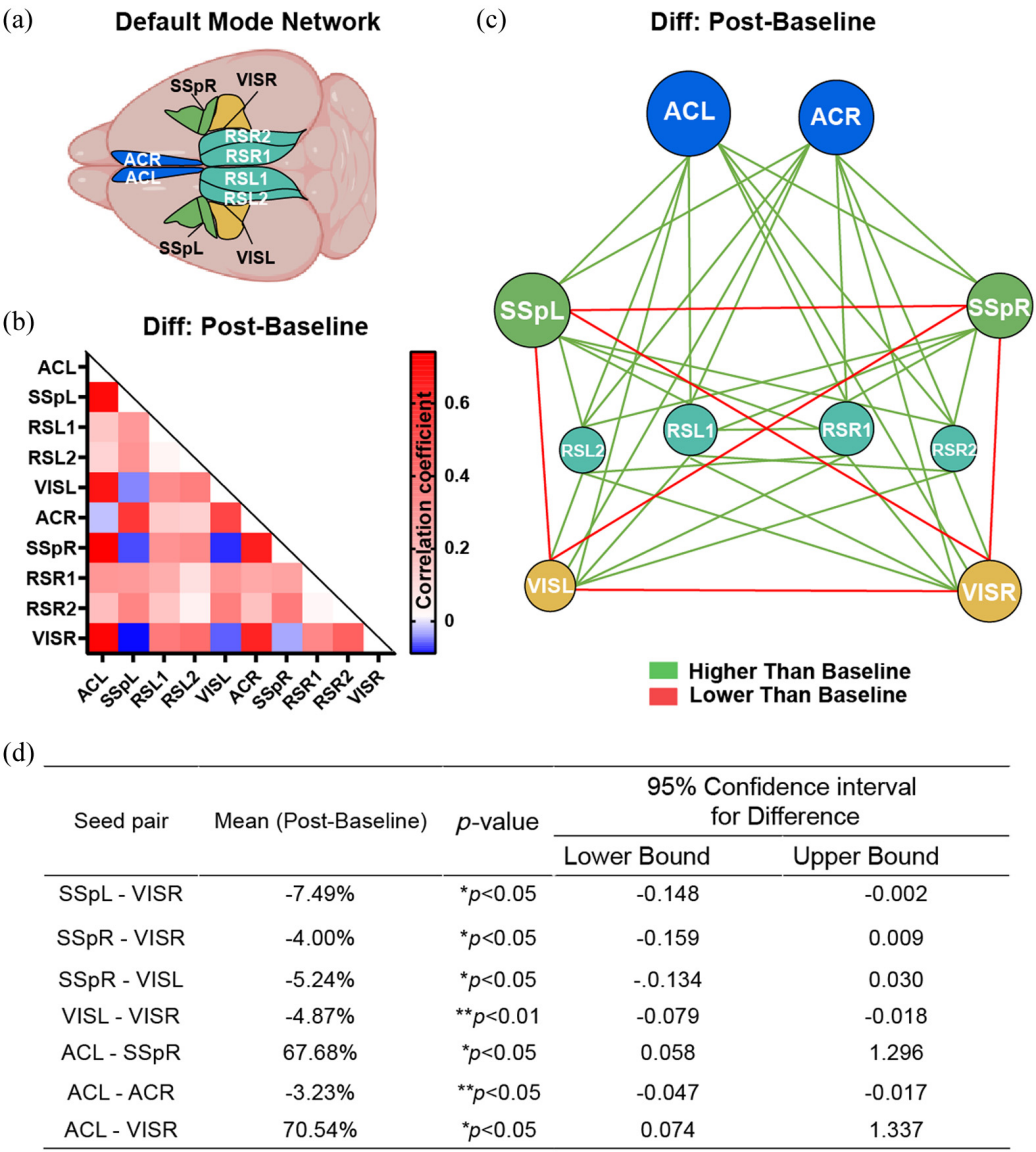
The DMN is altered after seizures. (a) Based on the Allen Brain Mouse Atlas, a schematic shows the DMN in the cortex. (b) The difference matrix was derived by subtracting the baseline matrix from the post matrix, which shows increased connectivity between the AC and other regions, the RS1 and other regions as well as that between the RS2 and other regions, while decreased connectivity between the left and right AC, the left and right SSp as well as that between the left and right VIS. In addition, functional connectivity is decreased between SSp and VIS. n=6. (c) The mean network node map of the DMN (threshold = 0.04). (d) Summary of seed-based interregional functional connectivity. n=6. *p<0.05, **p<0.01, ***p<0.001, paired two-tailed Student’s t-test. DMN, default mode network; SSp, primary somatosensory region; VIS, visual region.

DMN is thought to be essential for maintaining regular intrinsic brain activity and higher cognitive function connectivity.^27^ There is growing evidence of DMN abnormalities in patients with epilepsy.^28^^,^^29^ Consequently, we produced the difference matrix and the mean network node map to evaluate changes in the DMN after seizures [Fig. 7(a)].^29^ The results showed a general increase in the functional connectivity of the DMN [Figs. 7(b) and 7(c)], especially between AC and other regions as well as that between RS and other regions. Conversely, functional connectivity decreased between the left and right AC, the left and right SSp, as well as that between the left and right VIS. Moreover, there was a significant reduction in functional connectivity between SSp and VIS [Figs. 7(b)–7(d)]. These results suggest that DMN is affected after PILO-induced seizures.

## 4 Discussion

In this study, IOS and EEG were used to record the changes in brain activity and functional connectivity in a PILO-induced seizure model in mice. First, we found that brain activity increased dramatically during seizures but only partially recovered after seizures, with PT, RS, V1, and V2 being affected the most. Then, the connectivity between visual association regions (e.g., V1 and V2) and PT as well as RS decreased after seizures, whereas functional connectivity increased between M2 and most other regions as well as that between AC and most other regions. In addition, the DMN was altered after seizures with hyperconnectivity between AC and other regions as well as that between RS and other regions. In conclusion, these research findings provide evidence of seizure-induced impairment in functional brain networks, shedding light on the connection between such impairments and abnormal behavior post seizures.

Seizure is closely related to neurovascular coupling (NVC).^30^ During seizures, NVC increases energy supply by modulating active neurons to communicate with local blood vessels, causing them to dilate and increase blood flow, which in turn provides more hemoglobin and oxygen.^31^ Our results showed that during PILO-induced seizures, a large number of neurons discharged at high frequencies, signal intensity decreased, and hemoglobin contents increased, indicating that the energy supply rose, which may be regulated by NVC. Furthermore, studies have shown that vasodilation and increased blood flow after convulsions^32^ suggest that NVC is still active after seizures. In our study, after seizures, neurons no longer fired at high-frequency and hemoglobin levels increased compared to baseline, which is consistent with the observations in the ferret cortical epilepsy model.^33^ The increased hemoglobin content was not uniform across the cortical regions, with PT, RS, V1, and V2 regions showing significantly increased, suggesting that these regions may be more susceptible after seizures. Moreover, in contrast to the onset of seizures, the partial recovery of hemoglobin content after seizures may be because neurons no longer fire at high frequency, but non-neuronal cell activity still requires oxygen to provide energy.^34^

Subsequently, we calculated interregional connectivity, and despite a general decrease in spectral content and signal strength across all areas following PILO-induced seizures, connectivity changes between these regions exhibited both increases and decreases. According to the Pearson correlation formula, it is evident that connectivity is influenced not only by signal strength but also by the synchronization between two regions. In addition, other studies have also indicated a lack of consistency between changes in power spectra and connectivity.^35^ These observations collectively suggest that after epileptic seizures, connectivity changes between different areas vary. This variability may be associated with the formation of abnormal brain circuits, calling for further in-depth research.

Resting state networks can be altered after seizures,^36^ and these networks are essential for fundamental brain functions, such as attention, emotion, and cognition. These networks can be divided into two categories: functional networks for movement, sensation, and vision; and the network for higher-order brain operations, including the DMN.^37^ Our study also showed that functional cortical networks altered after PILO-induced seizures, with increased connectivity between the motor-associated regions (e.g., M2, MB) and other brain regions. The increased connectivity may explain the fact that seizures are often accompanied by limb jerking. Furthermore, we found the medial subnetwork changed after seizures, particularly decreased connectivity between PT and visual association regions (e.g., V1, V2). PT processes cortical signals from visual association regions that guides visual perception.^38^^,^^39^ Its decreased connectivity with visual association regions implied abnormal visual perception, which explains the visual hallucinations and visual illusions that often occur early in epilepsy.^40^^,^^41^ Another study demonstrated that neurons in the visual cortex of rats are damaged after convulsions,^42^ which could be responsible for the anatomical aspect of this decreased connectivity. In addition, the above changes have been observed in the chronic phase of the PILO model,^35^ suggesting the connectivity changes may be carried over into epilepsy formation or shared features of brain network damage changes in epilepsy. In conclusion, functional networks changes, particularly decreased connectivity between the PT and visual association regions in the medial subnetworks, may be an indicator for diagnosing seizures or even for predicting epilepsy.

In addition, most fMRI studies reveal impaired somatosensory networks in patients with temporal lobe epilepsy,^7^^,^^43^ which are greatly aligned with our findings, highlighting impaired sensory networks, particularly within the small medial subnetworks, potentially serving as seizure predictors. Inconsistently, our study revealed increased functional connectivity in the motor-associated cortex. This could result from compensatory responses or movement in the awake state.^44^

The DMN is also altered after seizures in our study and its essential for memory and mood function.^45^^–^^47^ AC and RS^48^ are crucial nodes in the DMN,^49^ with increased connectivity to other regions in our study. Diminished functional connectivity between AC and other regions has been reported to be associated with social disorders^50^ and anxiety behavior.^51^ The enhanced functional connectivity suggests that social and emotional impairment may not be apparent after seizures or even in the early stages of epilepsy. Consistently, clinical studies have confirmed that a single convulsive seizure did not affect patients’ social and emotional behaviors.^52^ Another key node, RS, receives input from V1, M2, and other cortical regions and plays a key role in integrating visual and spatial information, contributing to the formation of memory for spatial information.^53^ Our result suggests that memory function for spatial information is not impaired after seizures. It also has been reported that memory was not affected in the early period of epilepsy.^42^

Our study also found that PILO induces seizures early with only a partial decrease in connectivity in the DMN. Other studies showed that both animal models and patients during the chronic phase of epilepsy exhibited extensive DMN decreased,^54^^,^^55^ and behavioral manifestations in impaired spatial memory and social interaction.^8^^,^^56^ The differences in changes in connectivity in DMN during epilepsy compared to acute seizures may be due to the presence of extensive cortical damage, neuronal death, microglia activation, and astrocyte proliferation in the brain.^57^^,^^58^ However, increased connectivity in the DMN has been reported in the chronic phase in a KA model.^59^ This may be related to the anesthesia, which may induce hyperconnectivity in the DMN.^13^ Future research should elucidate the DMN changes at rest during epileptogenesis and the underlying pathophysiological mechanisms.

In contrast to previous studies that have explored alterations in intrinsic optical signal intensity within epilepsy models, our research represents a groundbreaking approach. We are the first to use IOS imaging of both hemispheres covering most of the cortical regions in a seizure model induced by PILO in head-fixing awake mice with a relatively non-invasive craniotomy. Previous studies have used intracranial injections of drugs, such as 4-AP,^60^ penicillin,^61^ or bicuculline,^33^ in mice to induce seizures. In the 4-AP and bicuculline models, the signal intensity was attenuated at the intracranial injection site but elevated in the surrounding region. However, diminished signal intensity in cortical regions, mainly concentrated near the midline, was observed in the penicillin-induced model and in our study. In addition, we also found that signal intensity was significantly reduced, especially in the PT and RS regions. Spectrogram analysis at 0 to 0.1 Hz indicated that low-frequency oscillatory signals in the cortex were suppressed after seizures. The above differences may be explained by the modeling method, anesthetic drugs, or intracranial injection. Intracranial injection of bicuculline and 4-AP directly induced excitability of localized neurons in the cortex. Bicuculline elevated neuronal excitability mainly by antagonizing GABA-A receptors,^62^ whereas 4-AP induced seizures mainly by blocking potassium ion channels.^63^ These methods are primarily employed in the study of epileptic waves propagation induced by topical application in the cortex. In this study, intraperitoneal injection of PILO mainly acted on cholinergic system. Its pathology is considered to initiate in the temporal lobe and extend to the cortex and thalamus.^64^ The PILO model is considered to be one of the optimal models to simulate patients with temporal lobe epilepsy in clinic and to observe cortical network changes during the awake state.

Our study also confirmed that the functional connectivity of mice at rest was significantly reduced under anesthesia. In addition, distinct anesthetic protocols have distinct effects on the functional connectivity of rodents.^13^^,^^65^ Thus, the awake state can more accurately simulate the changes in cortical functional connectivity at rest after seizures in the clinic. With high spatial resolution (<200  μm) and temporal resolution (<0.01  s),^16^ IOS can accurately identify all the regional variations (at least in the cortex covering by the giant craniotomy) and integrate with EEG to track seizures. However, our study also has some limitations. IOS can only detect cortical signals.^66^ Brain signals in subcortical structures like the hippocampus or thalamus that are considered crucial for the brain circuit of seizures should be assessed in future studies.^67^ Current studies of brain circuits often use GCaMP and iGluSnFR techniques with better temporal resolution and direct recording of neuronal signals,^68^ but the method is sensitive to brain mobility, and somatic signals can be contaminated by extrafoveal fluorescence.^69^ Importantly, these genetically encoded indicators can buffer essential messengers or neurotransmitters, which may result in abnormal brain activity.^70^

To conclude, our study methods are simple and sufficient to reveal a dynamic and prolonged alteration of functional connectivity in the resting state after seizures. In addition, it contributed to understanding the underlying circuit mechanisms behind brain dysfunction after seizures.

## Data Availability

The data and code that support the findings of this study are openly available at DOI: 10.6084/m9.figshare.23788164.

## Credit Author Statement

Conceptualization: LG and YX, and SC. Data curation: LG, XH, and ZH. Formal analysis: LG, HL, ZZ, YX. Investigation: LG, XH, ZH, CC, HL, ZZ, YX, JF, and YX. Project administration: LG and YX. Funding acquisition: HX, LZ, SC, YX. Supervision: YX, LZ. Writing - original draft: LG. Writing - review and editing: YX.
